# Stabilization of clay soils exposed to freeze-thaw conditions with waste Kevlar

**DOI:** 10.1371/journal.pone.0331597

**Published:** 2025-09-05

**Authors:** Rahim Kağan Akbulut

**Affiliations:** Technical Vocational School of Higher Education, Ataturk University, Erzurum, Türkiye; Builders Engineering College, INDIA

## Abstract

In recent years, the use of waste materials for soil stabilization has gained attention due to their environmental and economic advantages. Kevlar, a synthetic, high-strength fiber commonly used in telecommunications, becomes a significant source of industrial waste at the end of its service life. In this study, the potential utilization of waste Kevlar material for improving clay soils against freeze-thaw effects was investigated using computed tomography (CT) and scanning electron microscopy (SEM) imaging techniques. For this purpose, waste Kevlar was randomly mixed into two types of clay soils (CL and CH) at different dosages (0.05%, 0.25%, 0.5% and 1%) and fixed fiber length of 10 mm. The prepared samples were subjected to 2, 5, and 10 freeze-thaw cycles, after which their stress-strain behavior, peak stress values, and freeze-thaw resistance were evaluated. The experimental results indicated that the peak stresses increased in all cycles with the increasing of waste Kevlar content. Compared to the unreinforced soil, in CH clay reinforced with 1% Kevlar, peak stresses increased by approximately 23%, 26%, 59%, and 45% for 0, 2, 5, and 10 cycles, respectively. In the case of CL clay, the corresponding increases were approximately 76%, 43%, 49%, and 44%. These findings demonstrate the feasibility and sustainability of utilizing waste Kevlar as an effective reinforcement material to enhance the durability of clay soils against freeze-thaw conditions in geotechnical engineering applications.

## Introduction

Improving the engineering behavior of problematic clay soils in situ is considered both a cost-effective and practical solution, particularly in cold climate regions where seasonal freeze-thaw cycles significantly degrade soil performance. These cycles adversely affect key geotechnical properties, including strength, deformation, bearing capacity, and permeability. Consequently, civil engineering structures founded on clay soils—including irrigation channels, retaining walls, road and railway subgrades, embankments, and slopes—are often subjected to damage, resulting in repair costs amounting to millions of dollars. Thus, enhancing the freeze-thaw resistance of fine-grained soils has become a critical focus in geotechnical engineering.

Traditional soil stabilization methods typically involve the use of chemical agents or mechanical reinforcements [1–3]. However, recent years have seen growing interest in alternative techniques, such as the incorporation of nanomaterials [4–5], synthetic fibers like polypropylene (PP) and polyethylene (PE) [2,6–12], and various natural fibers [13–17]. These fibers are generally added in aligned or randomly distributed forms to improve soil strength, ductility, and durability. The literature on fiber-reinforced soils consistently reports two key outcomes: (1) fiber-reinforced soils exhibit higher shear strength than unreinforced soils, particularly under at high deformation levels, and (2) increasing the number of freeze-thaw cycles generally reduces the peak strength in both reinforced and unreinforced samples.

Concurrently, the accumulation of industrial and synthetic waste materials poses significant environmental and economic challenges. As a sustainable solution, the re-use of such waste products for soil stabilization and freeze-thaw enhancement has become a growing area of research [18–20]. Fiber reinforcement introduces complex interactions at the soil-fiber interface, involving mechanisms such as crack, bridging, tensile stress distribution, and interparticle bonding, all of which depend on the mechanical and physical properties of the fibers. Advanced imaging techniques, such as scanning electron microscopy (SEM), optical microscopy, and computed tomography (CT), have been instrumental in understanding these microstructural interactions [21–27].

Under freeze-thaw conditions, clayey soils are particularly vulnerable due to the expansion and contraction of pore water and the formation of ice lenses, which generate internal stress, cracking, and long-term strength degradation. To mitigate these effects, reinforcement fibers must possess high tensile strength, effective stress transfer capabilities, and strong adhesion with fine-grained soil particles. Randomly distributed fibers can help restrain deformation, delay crack propagation, and improve freeze-thaw performance by acting as tensile elements within the soil matrix [28].

Kevlar, a high-strength synthetic fiber widely used in telecommunications to protect fiber optic cables, is now produced in large quantities. When these cables become obsolete or damaged, Kevlar is discarded as industrial waste, despite its valuable engineering properties. In this study, Kevlar fibers obtained from decommissioned communication cables were repurposed for use as reinforcement in clay soils. This sustainable practice supports waste minimization and reduces dependency on virgin materials. By offering both environmental and economic benefits, the reuse of Kevlar aligns with sustainable development goals aimed at improving resource efficiency and minimizing ecological footprints.

The priority of this study is to highlight the reuse of waste Kevlar fibers as a sustainable reinforcement material to improve the freeze-thaw resistance of clayey soils, which has been poorly studied in the literature. Although previous studies have investigated the use of various synthetic and natural fibers, the behavior of Kevlar-reinforced clayey soils, especially under freeze-thaw cycling, remains largely unexplored. The main objective of this research is to evaluate the mechanical performance and microstructural behavior of Kevlar reinforced clay soils subjected to repeated freeze-thaw cycles. Therefore, two types of clay soils (CL and CH) were reinforced with Kevlar fibers at four different dosages (0.05%, 0.25%, 0.5% and 1%) and tested for stress-strain behavior, ultimate strength and freeze-thaw durability. Furthermore, CT and SEM imaging techniques were used to gain deeper insight into the internal structural changes and kevlar fiber-soil interactions. This study also aims to promote the reuse of industrial waste materials as an environmentally sound and technically effective method for soil stabilization.

## Materials and methods

### Clay soils

**Fig 1:**
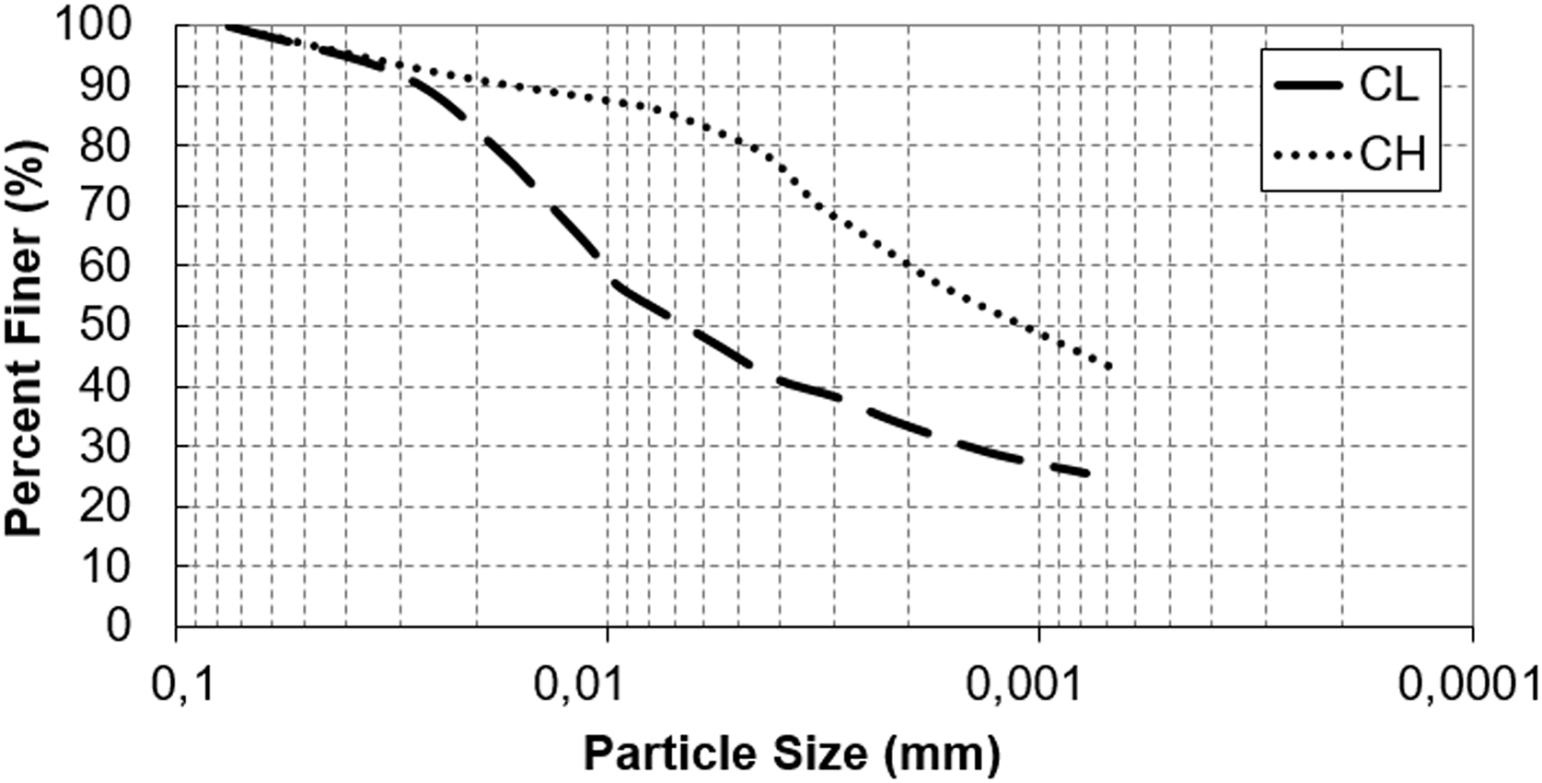
The particle size distribution curves of CL and CH clays.

Two different natural clay soils were obtained from the Erzurum region in eastern Türkiye, an area characterized by severe freeze-thaw cycles. Red and white clay samples were collected from a construction excavation site at a depth of approximately 1.0 meter to ensure minimal organic content and consistency. To ensure that the samples are representative of the field, three replicate samples were collected from different points (at least 10 meters apart) within each site and homogenized to form a representative sample for each clay type. The samples were air-dried, pulverized, and sieved through a No. 4 (4.75 mm) sieve to remove coarse particles, then oven-dried at 105 ± 5 °C for 24 hours. Based on Atterberg limits and grain size distribution curves (Fig 1), the red and white clay soils were classified as high plasticity clay (CH) and low plasticity clay (CL), respectively, according to the Unified Soil Classification System (USCS) [29]. The use of two distinct clay types aimed to evaluate the influence of waste Kevlar fibers across a range of plasticity conditions under freeze-thaw cycles. Some engineering properties of clay soil samples determined in laboratory conditions are presented in Table 1 [34].

**Table 1:** Some engineering properties of clay soil (CH, CL) samples.

Properties	CH	CL
Liquid Limit^1^, W_L_ (%)	120	36
Plastic Limit^2^, W_P_ (%)	30	24
Plasticity Index, I_P_ (%)	90	12
Specific Gravity, Gs	2.78	2.74
Clay Content, (%)	61.6	34.1
Optimum Water Content^3^, W_opt_ (%)	32	23
Max. Dry Unit Weight3, γ_kmax_ (kN/m^3^)	13.1	16.4
Unconfined Compressive Strength^4^,UCS, (kPa)	385	294
Soil Class (USCS)	CH	CL

^1^w_L_ [30], obtained from [31], ^2^ w_P_, obtained from [32], ^3^ according to [26], ^4^ according to [33].

### Waste Kevlars

**Fig 2:**
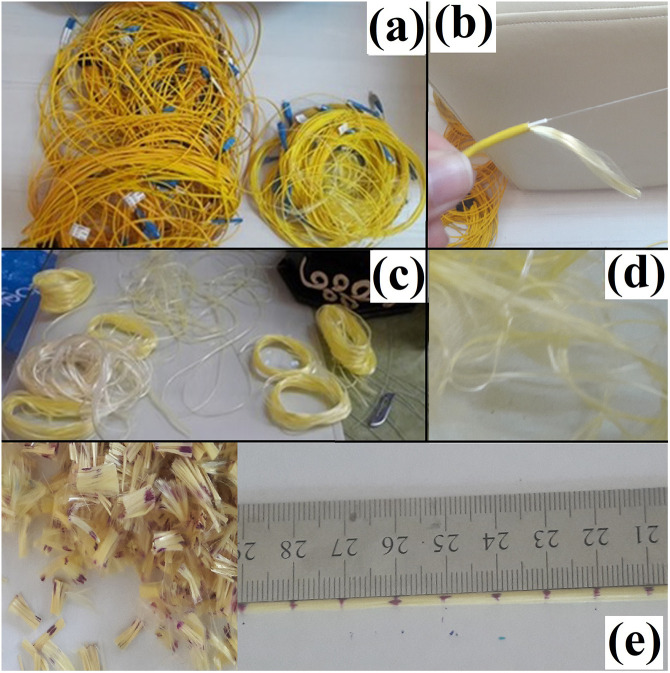
Obtaining waste kevlar (a-end-of-life fiber optic cable, b-kevlar, c-removal of kevlar from cable, d-waste kevlar, e-10 mm cut to length kevlar with scale-bar).

Kevlar, a synthetic braided material used to reinforce fiber optic cables, was repurposed for use in this study as a soil reinforcement material. Waste Kevlar was obtained by stripping it from decommissioned fiber optic cables (Fig 2a–2d). The recovered fibers were then cut to a uniform length of 10 mm using a scalpel and guide ruler (Fig 2e).

The Kevlar fibers were sourced from Türk Telekom Inc. and originated from fiber optic cables that had reached the end of their service life. The basic physical and mechanical properties of Kevlar used in this study are summarized in Table 2 [35] according to DuPont’s technical specifications. According to these specifications, Kevlar fibers maintain their tensile strength when exposed to various chemicals and environmental conditions.

**Table 2:** Some properties of kevlar used in tests [35].

Some properties	Values
Tensile Strength	Kevlar 49 fibers demonstrate values around 3,620 MPa, significantly surpassing the tensile strength of common carbon steels like AISI 1018, which ranges between 440–470 MPa
Melting Point	Reasonably good at withstanding temperatures, decomposes about 450°C
Ice Point	No degradation down to −196°C

To ultraviolet light causes discoloration in kevlars.

Kevlar retains its strength after prolonged exposure to dilute sulfuric acid and acetic acid at high temperatures.

### Preparation of waste kevlar reinforced samples and test program

In order to prevent agglomeration and to ensure homogeneity, waste kevlar was added gradually into the dry clay soil at the rates of 0.05%, 0.25%, 0.5% and 1% of the total dry weight of the reinforced soil, respectively during the preparation of the samples [7]. Water as much as optimum water content was little by little sprayed and mixed with dry clay-waste kevlar, then the mixtures are kept in a desiccator for 24 hours for the samples do not lose their water content. As can be understood from Table 3, freeze-thaw strength and UCS experiments were conducted at all Kevlar ratios and freeze-thaw cycle numbers for each group of experiments.

**Table 3:** Experimental program used in the study.

Test Group	Clay Type	Fiber Content (%)	Number of Freeze-Thaw Cyles	Conducted Experiments	
				F-T Resistance	UCS
**I**	CH	0	0-2-5-10	X	X
	CH	0.05	0-2-5-10	X	X
	CH	0.25	0-2-5-10	X	X
	CH	0.5	0-2-5-10	X	X
	CH	1	0-2-5-10	X	X
**II**	CL	0	0-2-5-10	X	X
	CL	0.05	0-2-5-10	X	X
	CL	0.25	0-2-5-10	X	X
	CL	0.5	0-2-5-10	X	X
	CL	1	0-2-5-10	X	X

**Fig 3:**
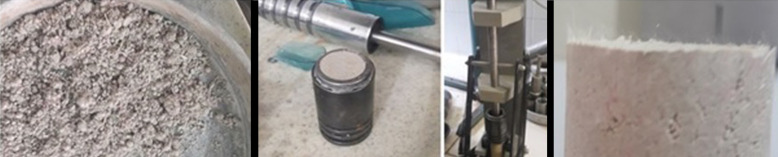
Preparation of samples (Homogeneous preparation of mixtures (left image), the sample inside the mold and sample ejector apparatus (middle images), distribution of waste kevlars in the sample (right image)).

Afterward, the mixtures were compressed with the Harvard Mini Compactor [36] device in five layers at standard proctor energy, and cylindrical samples with 32 mm diameter and 70 mm height were obtained (Fig 3). Harvard mini compactor is a standard static compaction test, and samples are frequently produced by this method (see for instance; [37–39]). These processes were repeated for all reinforced and unreinforced samples.

### Freeze – Thaw cycles

All freeze-thaw experiments were carried out in a programmable closed system freeze-thaw cabinet with a capacity of +60 °C and −25 °C. In accordance with the literature, the samples were left to freeze at −20 °C for 6 hours and to thaw at +25 °C for 6 hours [8]. These 12 hours in freezing and thawing is called one cycle. Based on regional climate data from the Turkish General Directorate of Meteorology [40], three cycle counts—2, 5, and 10—were selected to simulate typical field conditions in cold regions like Erzurum [33,41,42]. Samples were wrapped in aluminum foil coated with petroleum jelly and placed in the chamber for the full duration of each cycle without being removed. The freeze-thaw strength (FTG) (grain loss), calculated using Equation (1) [43]:


$$FTG\text{\textbackslash{} }(\%)=\frac{\left(IW-FTAW\right)}{IW}*\text{100}$$ (1)

Where:

IW: Initial weight of the sample

FTAW: Weights of the samples after freeze-thaw cycles.

Each test was conducted on three replicate specimens, and the average result was used for analysis.

### Unconfined compressive strength (UCS)

The unconfined compressive strengths of the samples were determined in accordance with ASTM D2166 [44]. Loading speed was taken as 0.8 mm/min in the experiments. The stress-strain relationship and peak stress values of all samples were obtained from the test results. The peak stress values that is equal to the highest values in the stress-strain relationship were recorded. In order to determine how much improvement is achieved, the peak stress values of the reinforced (waste kevlar) samples were normalized by proportioning them to the peak stress values of unreinforced clay samples and they are graphically presented in percentage.

### Imaging techniques: Computerized tomography (CT) and scanning electron microscope (SEM)

**Fig 4:**
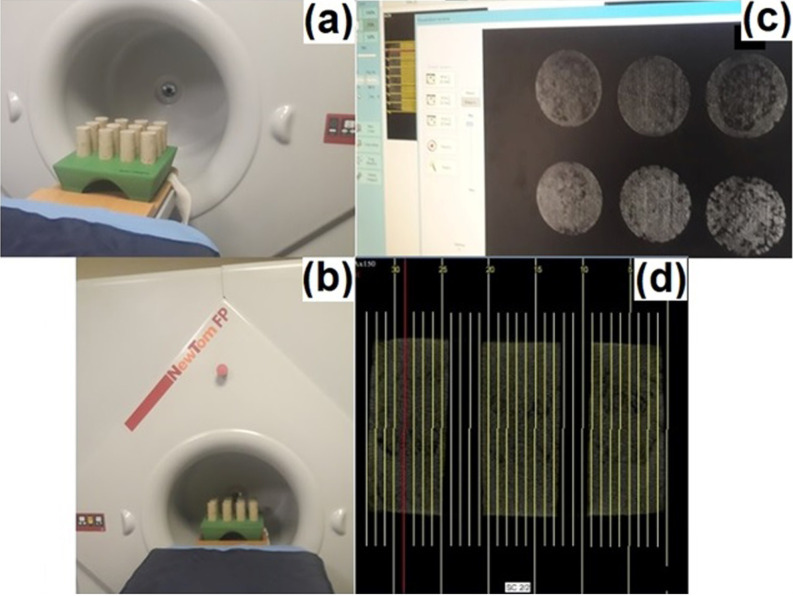
CT imaging unit (a- CT cabinet b- Sample tray, c- Cross section top views of slice samples, d- Longitudinal section of samples).

Both macro-size CT and micro-size SEM images employed to investigate the effects of freeze-thaw on reinforced and unreinforced samples. CT imaging unit is a “NewTom 3G Dental Volumetric” branded device with flat-panel that exists in Atatürk University Faculty of Dentistry. The device works with the conical beam technique at 110 kVp and a maximum 15mA. While the tube-flat panel detector system of the device rotating 360 degrees, it can obtain images at any degree in a cylindrical area of 13 cm height and 17 cm diameter. The scanning time of the device is 36 seconds (Fig 4a-4b)). With the tomography device procedure, the cross-section images of the samples were taken in 3 mm thick slices [24] (Fig 4-4c). Besides, CT images of the axial longitudinal sections of the samples were taken (Fig 4-4d). As a result, it can be said that numerous images of unreinforced samples and samples reinforced with 0.05% and 0.25% waste Kevlar were obtained by CT imaging technique.

Before SEM imaging, the samples were dried and coated with a thin layer of gold using a sputter coater to improve conductivity and prevent charging under the electron beam. SEM images were obtained at the Atatürk University Central Research Laboratory (DAYTAM) using a field-emission scanning electron microscope equipped with a Schottky field-emission electron gun and a four-segment backscattered electron detector (BSD). The instrument provides a resolution of 2.3 nm at 1 kV and 1.5 nm at 15 kV. A secondary electron detector enables magnifications up to 1,000,000 × . Elemental point analysis and two-dimensional mapping were performed using the EDX detector. Additionally, the In-Lens detector allowed for high-quality topographic imaging at lower accelerating volt-ages and shorter working distances. All observations were conducted under high-vacuum conditions to ensure optimal imaging quality.

## Results and discussion

### Freeze-thaw resistance and CT-SEM observations

**Fig 5:**
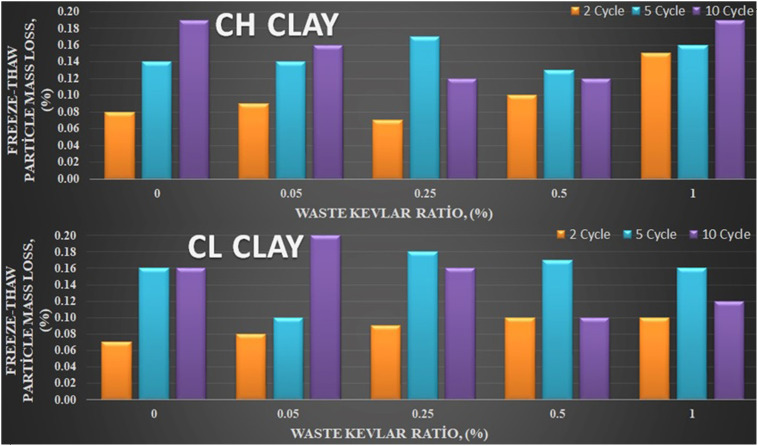
Freeze-thaw resistance change (a) CH Clay (b) CL Clay.

**Fig 6:**
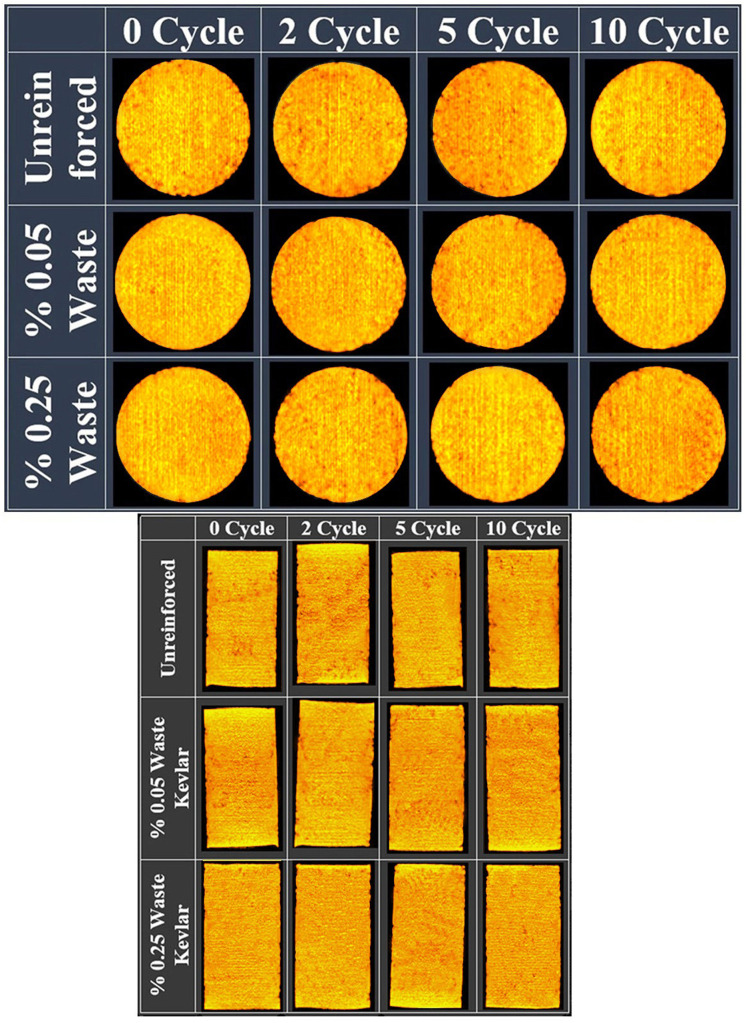
CT scan of CH clay (a- The top of sample’ 3 mm cross section image b- The middle of sample’ longitudinal section image).

**Fig 7:**
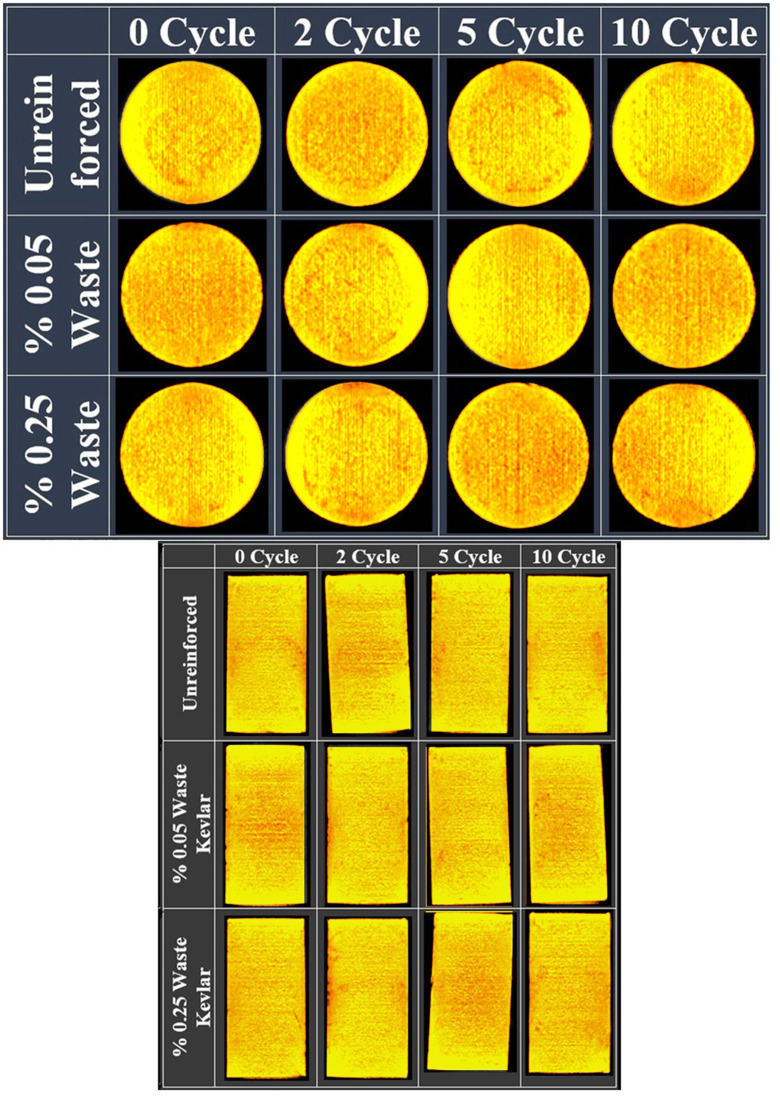
CT scan of CL clay (a- The base of sample’ 21 mm cross section image b- The middle of sample’ longitudinal section image).

**Fig 8:**
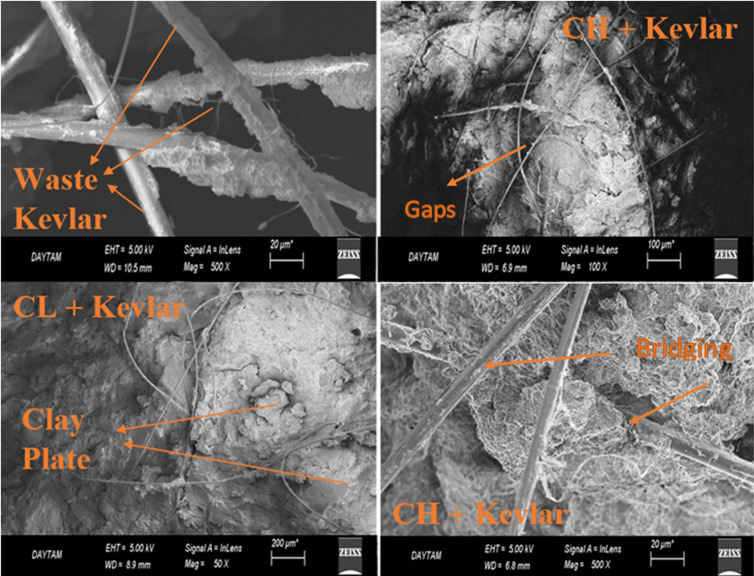
SEM images of CH and CL clay samples reinforced with waste kevlar and waste kevlar.

Graphs of freeze-thaw grain loss obtained with the help of equation (1) after 0, 2, 5, and 10 freeze-thaw cycles of clay soils with and without waste kevlar in different percent-ages (0.05%, 0.25%, 0.5%, and 1%) are shown in Fig 5. Cross-section and longitudinal CT images of CH and CL clays are shown in Fig 6 and Fig 7, respectively. While SEM images of Kevlar-reinforced samples are provided in Fig 8.

One can see from Fig 5 that freeze-thaw resistance generally decreases for CH and CL clays as the waste kevlar ratio in 2 and 5 freeze-thaw cycles increase to contrast while there is no such a tendency in unreinforced soil. On the other hand, in 10 cycles, it is observed that the freeze-thaw resistance increases in all addition rates [9]. This suggests that while Kevlar reinforcement has limited effect at low cycle numbers (2 and 5), its contribution becomes increasingly positive at higher cycles (10). This behavior—similar to that of polypropylene fibers—can be attributed to various mechanisms, including mineralogy, porosity, water movement, the effect of cycle numbers. From another point of view, this can be explained by the fact that the addition of Kevlar is effective at higher cycle numbers in terms of effective mobilization with the grains, increasing the voids between the grains and acting as a bridge effect in these areas. Specifically, Kevlar fibers may initially disturb the clay matrix at low cycles, causing localized expansion and micro cracks, particularly at lower reinforcement levels (0.05% and 0.25%). Over time, however, Kevlar fibers begin to act as bridging agents, filling voids and enhancing internal bonding. This structural integration helps to limit expansion and improve durability against freeze-thaw degradation, particularly after 10 cycles.

CT images were obtained to complement the SEM analysis and other experimental results, providing additional evidence of changes in the internal structure of the clay at the macro-level, strengthening the overall findings of the study. When Fig 6 and Fig 7 are evaluated together, it can be observed that the void ratio generally increases for up to 5 cycles in samples with unreinforced and reinforced soils with 0.05% and 0.25% amount of waste kevlar, whereas it decreases in 10 cycles [23]. In a similar fashion, it is seen that the void decreases relatively with the increase in the contribution of waste kevlar in the 0 cycle case. This indicates that the contribution of kevlar provides the binding feature among soil particles to a great extent [2,15] (Fig 8).

When the CT images of CL clay (Fig 7) and the freeze-thaw resistance (Fig 5b) are evaluated together, it is seen that the void ratio generally increases for the cases up to 5 cycles in both unreinforced and lightly reinforced samples (0.05% −0.25%), but decreases at 10 cycles. Additionally, comparison of the CT images for CH and CL clays (Figs 6 and 7) indicates that waste Kevlar fibers are more effective in CL clay. This can be attributed primarily to the lower water content and plasticity of CL clay, which results in lower water retention and makes it less vulnerable to freeze-thaw effects. Another important factor affecting this result is that they have different mineralogy. From this point of view, it is thought that the low swelling potential of CL clay (less water processing) may enable it to interact more effectively between kevlar and clay grains. In this context, Kevlar fibers contribute to the formation of mechanical bonds within the soil microstructure by effectively filling voids and enhancing overall stability.

Fig 8 reveals that waste Kevlar fibers possess numerous fine filaments attached to a robust central backbone, which appears superior in structure to the backbones of conventional polypropylene or polyethylene fibers. This unique morphology enables Kevlar to form a more effective bridging mechanism between clay particles, thereby enhancing interparticle bonding. The fine filaments extending from the main spine fill the void spaces within the soil matrix, contributing to increased ductility. As a result, the reinforced specimens are able to withstand greater stress, as the fishbone-like structure of the Kevlar fibers significantly increases the contact surface area compared to standard synthetic fibers. In other words, the fine, knotted structure of the Kevlar fibers forms close contact with surrounding clay particles, promoting tighter interparticle bonding. This enhanced binding effect increases the structural integrity of the soil matrix. Additionally, the high tensile strength of waste Kevlar helps to resist tensile stresses that may develop between particles during freeze-thaw cycles. The crumpled, net-like, herringbone morphology of Kevlar fibers limits particle displacement and inhibits the enlargement of voids. This interconnected fiber network further reinforces the soil by stabilizing the microstructure and distributing stresses more uniformly throughout the matrix.

When the experimental findings and imaging analyses are considered together, the literature identifies three types of voids between clay particles: macro, meso, and micro voids [27]. While macro-scale voids can be evaluated using CT and SEM imaging, the characterization of meso- and micro-scale voids requires more advanced and specialized imaging techniques. As previously noted, the addition of Kevlar fibers contributes to strength enhancement through a bridging effect. This mechanism is widely supported in the literature [11,27,45] and can be described as follows:

As axial pressure increases between clay particles, small-scale cracks begin to form, often initiated by water lenses that develop during freezing. Following freeze-thaw cycles, these cracks evolve into larger macro-pores, which eventually lead to the formation of fracture planes. The resulting deformation activates interlocking forces between soil particles. These forces are transferred to the Kevlar fibers, generating frictional contact at the soil-fiber interface. This interaction enhances the integrity of the soil matrix. Through its bridging effect, Kevlar helps establish a more cohesive and resistant matrix, supported by both mechanical interlocking and electrostatic interactions between the fibers and surrounding soil particles.

### Unconfined compressive strength

**Fig 9:**
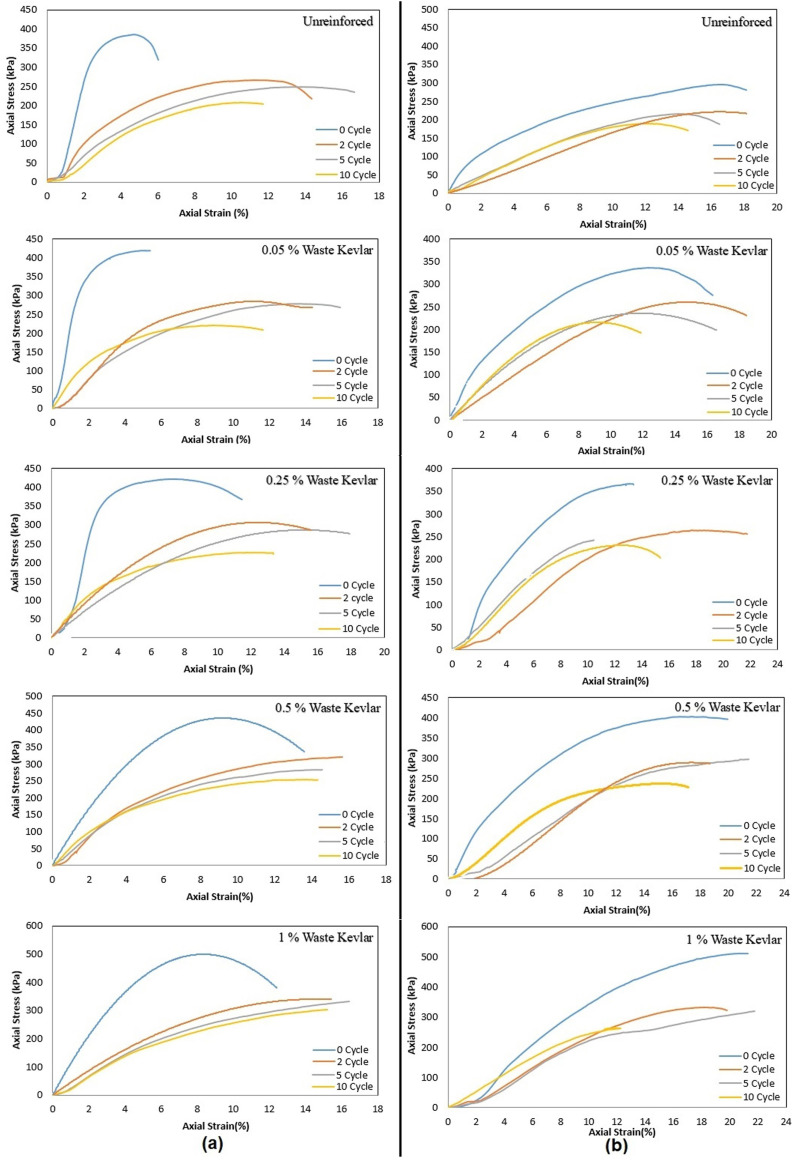
Axial stress-axial strain relationship (a- CH clay, b- CL clay).

**Fig 10:**
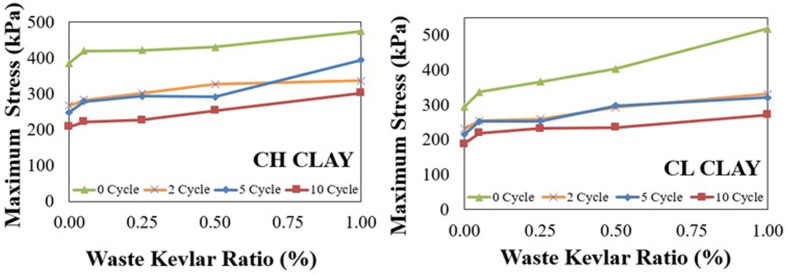
Relationship between maximum stress-waste kevlar ratios.

**Fig 11:**
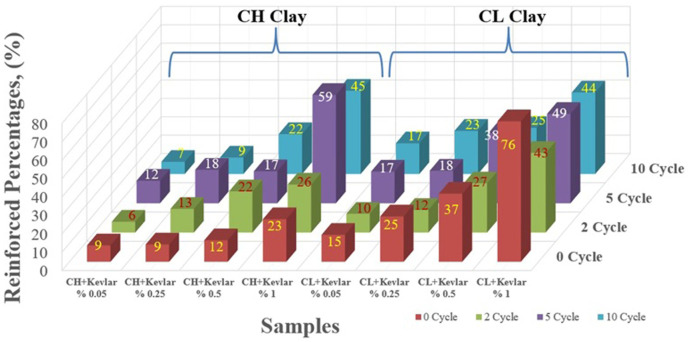
Reinforced percentages calculated based on unreinforced maximum stress values.

The axial stress-strain (σ-ε) relationships and corresponding peak stress values for samples with no reinforcement, and those containing 0.05%, 0.25%, 0.5%, and 1% waste Kevlar are shown in Fig 9 and Fig 10, respectively. To aid in the interpretation of these figures and to better visualize the improvement achieved through Kevlar reinforcement, a three-dimensional graph illustrating the normalized improvement percentages in relation to Kevlar content and freeze-thaw cycle numbers is provided in Fig 11.

For both CH and CL clays, Figs 9 and 10 demonstrate that maximum stress values and the initial tangent modulus decrease as the number of freeze-thaw cycles increases, regardless of whether the samples are unreinforced or reinforced with waste Kevlar [8]. This reduction in strength may be attributed to the development of capillary gaps within the soil matrix, potentially initiated by the presence of Kevlar fibers. CL clay is more resistant to freeze-thaw cycles due to its lower plasticity, while this effect was more pronounced in CH clay. Kevlar reinforcement increased the strength of both clay types in terms of UCS, but the increase was more pronounced for CL clay. On the other hand, while water is drained from the reinforced samples, it causes an increase in water content in locations where waste kevlar is found. Therefore, one may assert that the friction between waste kevlar and clay soil particles is relatively small which may cause a decrease the compressive strength of the soil like a capillary crack [31,32]. Nevertheless, Fig 10 clearly shows that maximum stress values increase with higher Kevlar content across all freeze-thaw cycle conditions (0, 2, 5, and 10). This strength gain is may likely due to the bridging action of Kevlar fibers, which effectively bind clay particles together, restrain volumetric expansion, and form a meshed structure around the grains, as illustrated in Fig 8 [13]. Additionally, the influence of lateral confinement during compaction should not be overlooked, as it may also contribute to the improved strength observed in Kevlar-reinforced specimens [46].

On the other hand, when Fig 10 is examined, in CH clay, the highest peak stress prior to freeze-thaw exposure is approximately 475 kPa with 1% Kevlar reinforcement. After 5 freeze-thaw cycles, this value decreases to around 394 kPa. For CL clay, these rates were determined approximately as 519 kPa at 1% contribution rate in 0 cycles and approximately as 331 kPa at the end of the freeze-thaw cycles with 1% waste Kevlar in 2 cycles.

In Fig 11, for 0, 2, 5 and 10 cycles, the reinforced percentages in CH clay that reinforced with 1% kevlar increased approximately 23, 26, 59, and 45% compared to unreinforced soil. Also, it increased about 76, 43, 49, and 44% for CL clay. To sum up it is understood from all the results that the samples reinforced with the waste Kevlar exhibit a ductile behavior compared to the unreinforced samples [45].

## Conclusions

This study investigated the freeze-thaw performance of CL and CH clay soils reinforced with recycled waste Kevlar fibers (0.05%, 0.25%, 0.5%, and 1%) under controlled laboratory conditions. Laboratory-prepared samples were subjected to 2, 5, and 10 freeze-thaw cycles and evaluated through stress–strain behavior, peak stress, and freeze-thaw resistance, supported by complementary imaging techniques (SEM and CT). The results reveal that freeze-thaw cycles significantly influence bearing capacity, durability, soil–water interactions, microstructure, and overall structural integrity of fine-grained soils. Addition of waste Kevlar fibers enhanced soil plasticity and strengthened weak interparticle bonds, leading to increased durability, particularly in subgrade soils exposed to repeated freeze-thaw cycles. The key outcomes of the study are summarized as follows:

- Effect of freeze-thaw cycles on mechanical behavior

Freeze-thaw resistance generally decreased for both CH and CL clays with the increasing amounts of waste Kevlar at 2 and 5 freeze-thaw cycles, compared to unreinforced soil. However, at 10 cycles, an improvement in freeze-thaw resistance was observed across all reinforcement levels. For both clay types, maximum stress and initial tangent modulus declined as the number of freeze-thaw cycles increased, regardless of reinforcement. In all cycles (0, 2, 5, and 10), the maximum stresses increased with the increase in the contribution ratio of the waste Kevlar. Samples reinforced with waste Kevlar exhibited a more ductile behavior compared to the unreinforced samples.

- Comparison between CL and CH clays, fiber content and ductility characteristics:

CL clay exhibited greater freeze-thaw resistance compared to CH clay; indicating that waste Kevlar fibers had a more pronounced reinforcing effect in CL clay. When CT images and freeze-thaw strength for CL clay are evaluated together, it is observed that the void ratio generally increased up to 5 cycles in both unreinforced samples and those reinforced with low Kevlar content (0.05% −0.25%), while it decreased in 10 cycles. Furthermore, as the Kevlar reinforcement ratio increased, the improvement percentages in peak stress and resistance consistently rose across all freeze–thaw cycle conditions compared to unreinforced soil.

The findings suggest that recycled Kevlar fibers can improve the mechanical behavior and durability of fine-grained soils, offering a sustainable and cost-effective alternative for subgrades in cold-climate infrastructure projects, such as roads and irrigation channels. This approach supports environmentally friendly geotechnical practices by utilizing waste materials for soil stabilization. The use of such recycled materials for mechanical stabilization is increasingly gaining attention in geotechnical research. To advance this research, the following recommendations are proposed:

- Future studies should investigate the use of various hard-to-dispose waste materials, including synthetic polymers such as polypropylene (PP) and polyethylene (PE) and natural fibers, for the stabilization of fine-grained soils.
- A wider range of freeze-thaw conditions (i.e., number of cycles, duration, and temperature) should be examined to evaluate the performance of treated soils over time.
- The effects of different drainage conditions and geotechnical parameters such as permeability, volumetric stability, and long-term durability should be evaluated.
- This study was conducted in a laboratory environment. However, the long-term performance of Kevlar reinforced soils should be verified at real field scale.
- Although waste material was used in the study, comprehensive cost-benefit analyses will further support the practical application of waste Kevlar in sustainable soil improvement techniques.

The results obtained in this study are based on experimental work in a laboratory setting, which may affect the transferability of the findings to field applications. However, some limitations of the study are listed below:

- The results were obtained under laboratory conditions, which are not influenced by the complex and variable factors of in-situ environments such as natural soil-water interaction, environmental temperature fluctuations, or actual loading conditions.
- The sample dimensions used may not fully represent the scale effects and mechanical responses of soils in field engineering applications.
- Freeze-thaw cycles in the study were applied under constant temperature conditions. In nature scenarios, such cycles may occur at varying temperature amplitudes depending on climatic changes, which could affect the behavior of the reinforced soils.
- Differences in water content and drainage conditions between laboratory and field applications may also introduce variability in performance.

In conclusion, the findings of this study demonstrate the potential of using waste Kevlar fibers as an effective and sustainable solution for soil stabilization in cold climate conditions. These results may encourage wider adoption of materials in geotechnical recycled engineering applications and contribute to the development of green construction practices.

## Supporting information

S1 Data used in the manuscript. (ZIP)
